# Patient-reported experiences and outcomes following two different approaches for non-surgical periodontal treatment: a randomized field study

**DOI:** 10.1186/s12903-021-02001-4

**Published:** 2021-12-15

**Authors:** Anna Liss, Jan L. Wennström, Maria Welander, Cristiano Tomasi, Max Petzold, Kajsa H. Abrahamsson

**Affiliations:** 1 Department of Periodontology, Institute of Odontology, The Sahlgrenska Academy, University of Gothenburg, Box 450, 405 30 Gothenburg, Sweden; 2 Clinic of Periodontology, The Public Dental Service, Region Västra Götaland, Medicinaregatan 12C, 413 90 Gothenburg, Sweden; 3 School of Public Health and Community Medicine, Institute of Medicine, The Sahlgrenska Academy, University of Gothenburg, Box 463, 405 30 Gothenburg, Sweden

**Keywords:** Non-surgical periodontal treatment, Patient-reported outcomes, Evidence-based practice, Effectiveness, Dental hygienists

## Abstract

**Context:**

The current report is part of a prospective, multi-center, two-arm, quasi-randomized field study focusing on the effectiveness in general praxis of evidence-based procedures in the non-surgical treatment of patients with periodontitis.

**Objective:**

The specific aims were to (i) evaluate patient-reported experience and outcome measures of treatment following a guided approach to periodontal infection control (GPIC) compared to conventional non-surgical therapy (CNST) and to (ii) identify potential predictors of subjective treatment outcomes and patient’s adherence to self-performed infection control, i.e. adequate oral hygiene.

**Methods:**

The study sample consisted of 494 patients treated per protocol with questionnaire- and clinical data at baseline and 6-months. The GPIC approach (test) comprised patient education for adequate oral hygiene prior to a single session of full-mouth ultra-sonic instrumentation, while the CNST approach (control) comprised education and instrumentation (scaling and root planing) integrated at required number of consecutive appointments. Clinical examinations and treatment were performed by Dental Hygienists, i.e. not blinded. Data were processed with bivariate statistics for comparison between treatment groups and with multiple regression models to identify potential predictors of subjective and clinical outcomes. The primary clinical outcome was gingival bleeding scores.

**Results:**

No substantial differences were found between the two treatment approaches regarding patient-reported experiences or outcomes of therapy. Patients’ experiences of definitely being involved in therapy decisions was a significant predictor for a desirable subjective and clinical outcome in terms of; (i) that oral health was considered as much improved after therapy compared to how it was before, (ii) that the treatment definitively had been worth the cost and efforts, and (iii) adherence to self-performed periodontal infection control. In addition, to be a current smoker counteracted patients’ satisfaction with oral health outcome, while gingival bleeding scores at baseline predicted clinical outcome in terms of bleeding scores at 6-months.

**Conclusions:**

The results suggest that there are no differences with regard to patient-reported experiences and outcomes of therapy following a GPIC approach to periodontal infection control versus CNST. Patients’ experiences of being involved in therapy decisions seem to be an important factor for satisfaction with care and for adherence to self-performed periodontal infection control.

*Registered at*: ClinicalTrials.gov (NCT02168621).

## Background

Periodontitis is one of the most common chronic diseases [1] with prevalence figures of about 40% in the Swedish adult population, whereas about 10% showing severe form of the disease [2]. Periodontitis may lead to considerable consequences for the individuals health, well-being and finances and also contribute to substantial healthcare costs for the society [3, 4]. The disease is characterized by inflammation, initiated by microorganisms, that results in loss of periodontal attachment [5]. The goal of non-surgical periodontal treatment is to establish and maintain periodontal infection control. The initial phase of the periodontal therapy comprises patient education, with focus on strengthening the patients motivation to adopt a more health-promoting behavior through individualized information and oral hygiene instructions, and subgingival instrumentation to disrupt biofilm and hard deposits. Since the patient’s adherence to self-performed infection control, i.e. adequate oral hygiene, is of crucial importance to reach and maintain desirable treatment results, great focus in the non-surgical therapy should be given to educational- and behavioral interventions [6, 7].

The Swedish national guidelines for adult dental care [8] recommend two different evidence based approaches for the initial non-surgical treatment phase of patients with periodontitis, that is; (i) a guided approach for periodontal infection control (GPIC) that has a pronounced initial focus on patient education, to establish adequate self-performed infection control, followed by a full-mouth ultra-sonic instrumentation at one session; or (ii) conventional non-surgical therapy (CNST) that comprise patient education and mechanical instrumentation (scaling and root planing) most often performed quadrant-wise at several sessions. The conclusions drawn from a recent systematic review, including 13 RCT-studies evaluating full-mouth versus quadrant-wise instrumentation, was that both methods are equally effective to achieve subgingival infection control in the initial non-surgical treatment of periodontitis patients [9]. It has been argued, however, that the GPIC approach has health-cost benefits since it is less time-consuming than CNST [10–12]. It has also been argued that the GPIC approach might be preferable in the initial instrumentation since many pockets respond positively to a less aggressive instrumentation and thus, that the healing obtained after initial instrumentation should “guide” which sites that are in need for further/more invasive treatment [11]. However, regardless of method for instrumentation, the success in periodontal therapy is highly dependent on the patient’s own efforts to control the infection. A careful patient education before instrumentation might increase the patient’s awareness of their role in therapy, with a positive impact on the patient’s adherence to adequate oral hygiene regiments as indicated in a previous study by Wennström et al. [12].

Evidence based results on treatment efficacy, as generated in RCT-studies, does not necessarily mean that the same results are reached when treatment is performed outside the strictly controlled research environment, i.e. general practice. For external validity and as pointed out in the Swedish national guidelines for adult dental care [8], effectiveness studies performed in general dental practice are therefore highly warranted. Moreover, even though evidence suggest that non-surgical periodontal therapy results in beneficial and meaningful outcomes for the patient, in terms of improved oral-health-related-quality-of life (OHRQoL) [13, 14], very little is known about how patients experience and value the benefits of treatment in accordance with the GPIC approach in comparison with CNST [9]. To include patient-reported outcome (PRO) measures in clinical trials, as a complement to “objective” clinical observations, are essential in order to evaluate the quality of care and to bring knowledge of importance to improve the quality of care [15, 16]. In addition, patient-reported experiences of the care (such as to receive clear information and to be involved in therapy decisions) and benefits of therapy (in terms of self-reported health outcomes) are closely linked to each other as well as to adherence to treatment recommendations and objectively measured health outcomes, that has been demonstrated in studies performed in medical care settings [17].

The current report is part of a clinical field study focusing on the effectiveness of evidence-based non-surgical treatment of patients with periodontitis. The specific aims were to:

(i) Analyze patient-reported experience and outcome measures of treatment following the GPIC approach to periodontal infection control compared to CNST,
(ii) Identify potential predictors of subjective treatment outcomes and patient’s adherence to self-performed infection control.

It was hypothesized that patients treated in accordance with the GPIC approach, with its pronounced initial focus on patient education, will show better adherence to self-performed periodontal infection control than those who received CNST.

## Methods

### Study design

**Fig. 1:**
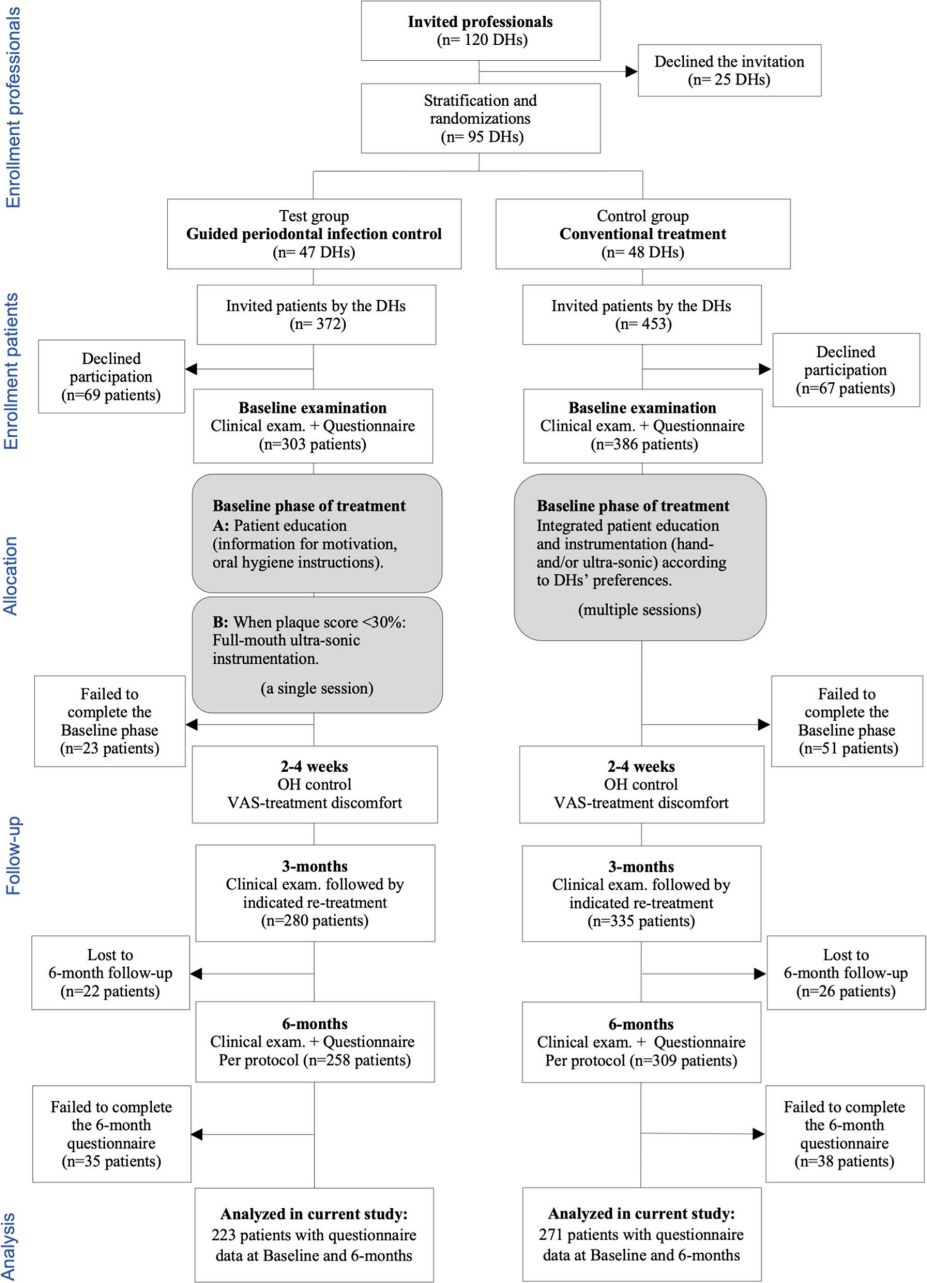
Study flow chart on the progress through the different phases; Enrollment, Allocation to Intervention, Follow-up and Analysis

Data analyzed in the present study derived from a prospective, multi-center, two-arm, quasi-randomized, field study focusing on the effectiveness in general praxis of evidence-based procedures in non-surgical treatment of patients with periodontitis. The study protocol was evaluated and approved by the Regional Ethical Review Board, Gothenburg, Sweden (Dnr: 288–13) and registered at ClinicalTrials.gov (NCT02168621). All study procedures were performed in accordance with relevant ethical principles and guidelines. In addition, Consort guidelines for reporting Clinical Trials [18] were carefully considered and followed when deemed as relevant with regard to the current field study. A flow-chart of the study is shown in Fig. 1.

### Professionals—dental hygienists

One-hundred-and-twenty Dental Hygienists (DH), who regularly treated patients with periodontitis, were invited to take part in the study and 95 DHs working at 59 different public dental clinics in the region of Västra Götaland (VG), Sweden, accepted the invitation (Fig. 1). The same DH who treated the study patient also performed the clinical examinations and consequently, the examiner was not blinded. Initially, the DHs participated in a one-day education and training for the study including Good Clinical Practice in research and a calibration session of clinical assessments. Based on the clinic at which they worked, the DHs were stratified and then randomized to the treatment protocol (test or control) by the second author (JW) and with the use of a computer software. The rationale behind this quasi-randomized procedure was to achieve a balanced distribution in the test and control group with regard to DHs working at clinics representing areas with different socio-demographic characteristics within the region of VG, which is one of the largest regions in Sweden representing both metropolitan, urban and rural areas. For quality control, site visits were carried out repeatedly during the study period by a project coordinator/monitor.

### Study population of patients

The study sample consisted of patients ≥ 30 years old, having at least 18 teeth and diagnosed with periodontitis (at least 5 teeth with probing depth ≥ 5 mm and bleeding on probing at proximal sites; i.e. generalized moderate to severe periodontitis, corresponding to at least stage 3 with regard to the new classification system [19]. Moreover, the study patients should have Swedish language knowledge and a general health condition that allowed participation in the study. Patients were recruited consecutively between June 2014 and December 2017. Following information verbally and in written about the study, voluntariness and confidentiality, all patients signed an informed consent before entering the study. Each DH recruited at least 6 patients.

### Treatment protocols

The test protocol—*‘Guided periodontal infection control’* (GPIC)—had a pronounced initial focus on patient education to establish an efficient oral hygiene (full-mouth plaque score < 30%) prior to initiation of the subgingival mechanical instrumentation. The mechanical debridement was carried out at a single session of full-mouth ultrasonic instrumentation [11].

The control protocol—*‘Conventional non-surgical treatment’* (CNST)—comprised patient education and mechanical instrumentation (scaling and root planing) integrated at required number of consecutive appointments, as judged by the DH and in accord with standard established routines of treatment, i.e., “business as usual”.

Two to four weeks after completion of the initial, baseline phase of treatment patients in both groups were scheduled for oral hygiene control and re-instruction if needed. Subsequently, a follow-up visit for oral hygiene control and re-instrumentation if indicated was scheduled in both groups at 3-months, and further at 6-months for clinical examination and evaluation (see Fig. 1).

### Data collection

#### Patient-reported data—questionnaires

Questionnaires were distributed at baseline and at 6-months. Information about back-ground characteristics (gender, age, education and smoking habits) were collected, as well as treatment related experiences and outcomes, oral hygiene behaviors and oral hygiene related self-efficacy beliefs (i.e. confidence in performing a specific behavior). The questionnaires were to be answered by the patient in the waiting room in conjunction with, but before, treatment and returned in a sealed envelope. The questionnaires were developed based on researchers expertise and on knowledge generated in previous studies, including questions from established scales, as described in details below. Prior to the study, the questionnaires were presented to a group of 8 patients under treatment at a clinic of periodontology and based on their comments some minor corrections were made to increase readability and understanding.

#### Patient-reported experience measures

At follow up 2–4 weeks after initial therapy, information was collected about the degree of treatment and post-treatment pain/discomfort experienced using a visual analogue scale (VAS) (100 mm, with no pain/discomfort and worst pain/discomfort as end words (see Wennström et al. [12]).

In addition, at the 6-month follow up the patients responded to questions about how they had experienced the communication with the DH during therapy and whether they were as involved in treatment decisions as they wanted. They were also asked about the degree of satisfaction with the treatment provided by the DH and if they considered the treatment worth the cost in terms of time, money and efforts. A 4-point response scale was used from the best experience/satisfaction to the worst/not satisfied at all. The questions were formulated based on knowledge generated in qualitative interview studies [20, 21] and on questions used in previous treatment studies [22] involving patients with periodontitis.

#### Patient-reported oral health measures

Self-perceived oral health was assessed at baseline and at 6-months with the global question *‘How satisfied are you with your oral health?’* In addition, at the 6-month evaluation the patients were asked *‘How would you compare your satisfaction with oral health after treatment with the way it was before treatment?’* The questions were answered on a 5-point response scale from very satisfied/very much better to very dissatisfied/worse [22].

The questionnaires at baseline and 6-months also contained The General Oral Health Assessment Index (GOHAI) (Swedish version; Hägglin et al. [23]). The GOHAI was chosen based on the results of a previous study suggesting this instrument as suitable for patients with periodontal disease [24]. The GOHAI is originally a 12-item questionnaire where patients were asked if during the past three months they had experienced any of the oral health related problems listed, with the main question *‘How often have you…because of your teeth/gums?’* For the current study 8 of the items were selected based on the results of a previous study involving a Swedish patient sample with periodontitis [14]. The response rate is on a five-point Likert scale (1 = always, often, sometimes, seldom, never = 5), given a range in the current study of 8–40 points, with higher scores indicating less problems, i.e. better OHRQoL. For two of the items, i.e. item related to ‘eating without discomfort’ and ‘satisfied with appearance’, a reversed coding was used.

#### Oral hygiene behaviors & Oral hygiene related self-efficacy

Frequency of tooth brushing, interdental cleaning and time (minutes) spent on daily oral hygiene procedures were assessed at baseline and 6-months. In addition, the patients’ self-efficacy for interdental cleaning was assessed at the 6-month follow-up, using a modified Swedish version by Jönsson et al. [25] of the original Self-Efficacy Scale [26]. The instrument used consisted of five statements concerning the person’s confidence to perform interdental cleaning in different taxing situations, with the main question formulated *‘How confident are you that you clean your interdental surfaces in the following situations?’* A six-point scale was used with scores from 0 = ‘absolutely confident to not clean’ to 5 = ‘absolutely confident to clean’, giving a range from 0–25 with higher scores consequently indicating stronger self-efficacy beliefs.

#### Clinical data

In order to evaluate the patient’s efforts regarding oral hygiene, i.e. adherence to self-performed infection control, bleeding (BI) and plaque (PI) scores [27] were assessed at baseline (before treatment) and at 6-months. BI (primary outcome) was assessed as present (1) or absent (0) following superficial probing at four sites on all teeth (mesial, buccal, distal and lingual). Likewise, presence (1) or absence (0) of dental plaque (PI) were detected by means of a probe on the tooth surfaces and recorded at four sites on all teeth in the 1^st^ and 3^rd^ jaw quadrants.

### Data analysis

Descriptive statistics were presented including frequency distributions, mean values and confidence intervals (95% CI). For categorical data, Chi-square analysis/Fishers exact test was used to test for differences between groups. Independent samples *t-*test was used for comparison between treatment groups for continuous data. The internal consistency and reliability of the GOHAI and Self-efficacy scales were assessed with Cronbach´s alpha. Finally, multiple logistic and linear regression analyzes were performed to identify potential predictors of subjective treatment outcomes and patient’s adherence to self-performed periodontal infection control. Logistic regression models were used for two different dichotomized subjective outcomes; (Model I) Patients’ overall satisfaction with the treatment, i.e. that treatment was worth the cost in terms of time, money and efforts, and (Model II) Patients’ satisfaction with oral health outcome after therapy, compared to the way it was before treatment. Further, a linear regression model was used to analyze; (Model III) Patients’ long-term adherence to periodontal infection control as determined by proximal BI scores at 6-months. The explanatory variables included in the multiple models were chosen based on theoretical assumption of potential associations with the outcome variables and preceding correlation analyses. SPSS (The Statistical Product Service Solution, version 26) was used for data management. Statistical significance was set as *p-*value < 0.05.

## Results

Out of the original sample of 567 patients treated per protocol, 73 patients (test, n = 35; control, n = 38) failed to complete the questionnaires at both baseline and at 6-months examination and were excluded from the current study. Hence, the current study sample consisted of 494 patients (test, n = 223; control, n = 271) treated per protocol and with questionnaire data available both at baseline and at 6-months follow-up (Fig. 1). A missing value analysis revealed that the 73 excluded patients were somewhat younger than the study sample (mean age 50 and 54 year; *p* < 0.01) but did not differ with regard to other baseline characteristics (number of teeth, gender, education and smoking habits).

Characteristics of the current study sample are shown in Table 1. No significant differences between the test and control groups were found at baseline with regard to mean age, gender, education or smoking. The mean number of teeth in the test and control group was 25.9 and 26.5, respectively (*p* < 0.01).

**Table 1:** Characteristics of the study sample at baseline; mean ± standard deviation and %. (total number of participants n = 494; test n = 223 and control n = 271)

	Test	Control
No. of teeth	25.9 ± 2.4	26.5 ± 2.0
Mean age	54.8 ± 11.2	53.6 ± 12.4
Gender, %		
Female	45.3	44.6
Male	54.7	55.4
Education, %		
Elementary school	23.4	17.4
High school	47.7	47.8
University	28.8	34.8
Smoker, %		
Current	24.2	23.8
Previous	35.5	35.9
Never	40.3	40.2

### Validity and reliability of questionnaires

The internal drop-out in the questionnaires was generally low (0–3.5% in almost all separate items). A correlation analysis between two items that were meant to reflect a similar content (self-rated oral health and satisfaction with oral health, respectively) indicated acceptable internal consistency reliability (rho = 0.71, *p* < 0.001). The Cronbach’s alpha for the GOHAI scale was 0.70 and for the Interdental Self-efficacy scale 0.90, also indicating acceptable to good internal consistency.

### Patient-reported experiences of treatment (PRE)

The results of PRE are shown in Table 2. The experiences of pain/discomfort related to therapy was rated in the lower range of the VAS in both the test and control group and with no significant differences between the groups.

**Table 2:** Patient-reported experiences of treatment; mean (95% CI) and % (test n = 223 and control n = 271)

	Test	Control	*p *value
*2–4 weeks follow-up*			
VAS^†^			
Discomfort during treatment	26.7 (23.4–30.0)	23.2 (20.3–26.1)	0.120^‡^
Discomfort post treatment	17.6 (14.8–20.3)	15.1 (12.9–17.2)	0.163^‡^
Sensitive root surfaces post treatment	12.4 (9.9–14.9)	12.0 (9.8–14.1)	0.782^‡^
*6-months follow-up*			
How did the communication between you and your DH work?			
Very well	90.0%	85.1%	0.149
Rather well	10.0%	14.5%	
Less well/poorly	-	0.4%	
Have you been as involved as you wish in treatment?			
Definitely	59.5%	62.1%	0.515
Mostly	38.2%	34.9%	
Doubtful/not at all	2.3%	3.0%	
How satisfied are you with the periodontal treatment the DH provided?			
Very satisfied	86.4%	84.7%	0.435
Rather satisfied	13.1%	15.3%	
Dissatisfied/very dissatisfied	0.5%	-	
Was the periodontal treatment worth the cost, in terms of time, money and efforts?			
Definitely	63.3%	63.3%	0.039
Mostly	32.1%	27.0%	
Doubtful/not at all	4.6%	9.7%	

Fisher′s exact test and Independent Samples *t-*test^‡^. ^†^Visual Analog Scale (0–100 mm)

A majority of the patients, in both treatment groups, had a positive experience of the communication with the DH during treatment. Most patients also experienced that they had the opportunity of being as involved as they wanted in therapy decisions and reported an overall high degree of satisfaction with the treatment provided, with no statistically significant differences between the groups. In both treatment groups, 63% of the patients considered that the treatment was definitely worth the cost in terms of time, money and efforts, while about 5% of the patients in the test group and 10% in the control were doubtful or did not at all agree that the treatment was worth the cost and efforts (*p* < 0.05; Table 2).

### Patient-reported outcome of treatment (PRO)

The results of PRO in terms of self-perceived oral health and aspects on OHRQoL are shown in Table 3. The level of satisfaction with oral health increased from baseline to 6-month evaluation in both treatment groups. About 77% of the patients valued their satisfaction with oral health after therapy as very much better or much better than before treatment, with no significant differences between the groups.

**Table 3:** Patient-reported satisfaction with oral health and perceived impact of oral health on daily life & well-being; % and mean (95% CI). (test n = 223 and control n = 271)

	Baseline			6-months		
	Test	Control	*p *value	Test	Control	*p *value
How satisfied are you with your oral health? %						
Very satisfied	2.7	1.1	0.339	6.3	8.9	0.503
Satisfied	30.8	34.1		48.4	44.2	
Neither satisfied nor dissatisfied	45.7	39.3		33.5	36.8	
Dissatisfied	19.0	23.7		8.6	8.6	
Very dissatisfied	1.8	1.9		3.2	1.5	
How would you compare your satisfaction with your oral health after treatment with the way it was it before treatment? %						
Very much better	–	–		27.6	29.9	0.808
Much better	–	–		49.3	47.8	
Slightly better	–	–		18.1	19.0	
No difference	–	–		4.5	3.4	
Worse	–	–		0.5	–	

The general oral health assessment Index^†^ (GOHAI)	Baseline			6-months		
During the past three months: ‘How often have you… because of your teeth and gums?’	Test	Control	*p *value^‡^	Test	Control	*p *value‡
‘Trouble biting or chewing food’	4.5 (4.4—4.6)	4.5 (4.4—4.6)	0.662	4.6 (4.5—4.7)	4.6 (4.5—4.7)	0.986
‘Eating without discomfort’	3.8 (3.5–4.0)	3.9 (3.7—4.1)	0.210	3.9 (3.7—4.1)	3.9 (3.7—4.1)	0.835
‘Limit contact with others’	4.8 (4.7—4.9)	4.8 (4.7—4.8)	0.908	4.8 (4.7—4.9)	4.8 (4.7—4.9)	0.946
‘Felt pain or discomfort’	3.8 (3.7–4.0)	3.7 (3.6—3.8)	0.108	4.0 (3.9—4.1)	4.0 (3.9—4.1)	0.552
‘Satisfied with appearance’	3.3 (3.1—3.4)	3.2 (3.0—3.3)	0.422	3.5 (3.3—3.6)	3.6 (3.5—3.8)	0.157
‘Worried or concerned’	3.4 (3.3—3.6)	3.4 (3.3—3.6)	0.919	3.6 (3.5—3.8)	3.7 (3.5—3.8)	0.935
‘Nervous or self-conscious’	3.9 (3.8—4.1)	3.9 (3.8—4.0)	0.858	4.0 (3.9—4.1)	4.1 (3.9—4.2)	0.561
‘Uncomfortable eating with others’	4.7 (4.7—4.8)	4.7 (4.6—4.8)	0.513	4.8 (4.7—4.9)	4.7 (4.7—4.8)	0.726
Mean; total GOHAI score	32.1 (31.5—32.8)	32.1 (31.5–32.6)	0.821	33.2 (32.6—33.8)	33.3 (32.8—33.8)	0.795

Fisher′s exact test and Independent Samples *t-*test^‡^. ^†^A five-point scale was used with scores from 1 = Always, to 5 = Never; range 8–40 with higher scores indicating less problem, i.e. better OHRQL

The change in mean scores of the GOHAI scale from baseline to 6-months was statistically significant for both groups (*p* < 0.001), without any significant differences between the groups, indicating less problems related to oral health after therapy, i.e. better OHRQoL. Looking at separate items of the GOHAI scale, there were no statistically significant differences between the treatment groups at any time point. In both groups, the items indicating the least problems at both baseline and 6-months were ‘limit contacts with others’ and ‘uncomfortable eating with other people’ (Table 3).

### Oral hygiene behaviors & Oral hygiene related self-efficacy

As shown in Table 4 most of the patients in both groups reported that they brushed their teeth ≥ 2 times/day at baseline as well as at 6-month follow up, without any statistically significant differences between groups. The frequency of daily interdental cleaning increased during therapy, with a somewhat more pronounced improvement in the control group but with no statistically significant differences between the groups in this aspect neither at baseline nor at the 6-months follow-up. The time spent on oral hygiene procedures also increased from approximately 8 to 10 min/day with no significant differences between the treatment groups. In addition, no significant differences were found between the treatment groups at 6-months regarding self-efficacy for inter-dental cleaning, i.e. confidence to perform interdental cleaning in different taxing situations. The lowest rated self-efficacy for interdental cleaning was related to the situation of ‘having a head-ache or not feeling well’ followed by ‘when tired in the evening’. The highest rated self-efficacy score was related to the confidence to perform interdental cleaning even though ‘not going to the DH/dentist in the near future’ (Table 4).

**Table 4:** Self-reported oral hygiene habits at baseline and 6-months and self-efficacy beliefs for interdental cleaning at 6-months; % and mean (95% CI). (test n = 223 and control n = 271)

	Baseline			6-months		
	Test	Control	*p *value	Test	Control	*p *value
Frequency of tooth brushing, %						
≥ 2 times a day	88.7	85.1	0.483	88.8	86.7	0.368
Less often	11.4	14.9		11.2	13.3	
Frequency of interdental cleaning, %						
Daily	40.2	41.0	0.062	58.1	63.6	0.553
3–5 times a week	23.7	14.7		27.0	23.4	
Less often or never	36.1	44.4		14.9	13.0	
Time spent on oral hygiene, min/day	8.0 ± 4.2	8.0 ± 5.2	0.923^‡^	9.5 ± 5.5	9.7 ± 5.9	0.785^‡^
Inter-dental cleaning self-efficacy^†^						
*‘How confident are you that you clean your proximal surfaces in the following situations?’*						
When you are tired in the evening	–	–		3.1 (2.9–3.3)	3.2 (3.0–3.3)	0.533^‡^
When you are not going to the DH/dentist in the near future	–	–		3.9 (3.7–4.0)	3.9 (3.7–4.0)	0.904^‡^
When you are on holiday	–	–		3.7 (3.5–3.8)	3.7 (3.6–3.9)	0.664^‡^
When you have a lot of work	–	–		3.6 (3.4–3.7)	3.6 (3.4–3.7)	0.888^‡^
When you have a headache or feel ill	–	–		3.0 (2.8–3.1)	3.1 (2.9–3.2)	0.448^‡^
Mean; total self-efficacy score	–	–		17.2 (16.5–17.8)	17.4 (16.8–18.0)	0.607^‡^

Fisher′s exact and Independent Samples *t-*test^‡^. ^†^A six-point scale was used with scores from 0 = Absolutely confident to not clean, to 5 = Absolutely confident to clean; range 0–25 with higher scores indicating better self-efficacy

### Patient’s adherence to self-performed periodontal infection control

Table 5 shows the data for BI (primary clinical outcome) and PI scores at baseline and 6-months. BI score was most pronounced at proximal surfaces with a mean score at baseline of 47% and 49% for the test and control group, respectively, with no significant differences between the groups. In both groups, a statistically significant decrease in BI scores was shown from baseline to 6-months follow up (*p* < 0.001) with no significant differences between the groups. At the 6-month examination the mean proximal BI score was about 20% in both groups (Table 5). Furthermore, at the 6-month evaluation about 35% of the patients in the test group and 38% in the control group presented with a BI score of ≤ 10% at proximal sites. Still, about 6% in the test and 9% in the control group remained with a poor infection control after therapy (proximal BI scores ≥ 50%).

**Table 5:** Bleeding and Plaque scores at baseline and 6-months; mean % (95% CI). (Bleeding score: test n = 198 and control n = 257; Plaque score: test n = 197 and control n = 237)

	Baseline			6-months		
	Test	Control	*p *value	Test	Control	*p *value
Bleeding score, %						
All surfaces	30.4 (27.4—33.5)	32.9 (29.9—35.8)	0.266	12.5 (11.0—14.1)	13.1 (11.4—14.9)	0.636
Proximal	46.6 (42.6—50.5)	48.9 (45.7—52.1)	0.367	20.2 (17.8—22.6)	19.6 (17.4—21.9)	0.726
Plaque score^†^, %						
All surfaces	34.0 (30.6—37.5)	44.6 (41.2—47.9)	< 0.001	14.6 (12.9—16.3)	20.8 (18.2—23.3)	< 0.001
Proximal	50.1 (45.6—54.7)	62.6 (58.6—66.6)	< 0.001	21.5 (18.7—24.3)	28.8 (25.3—32.2)	0.001

^†^ Plaque scores in quadrant 1 and 3. Independent Samples *t-*test

A statistically significant difference in PI scores was found between the groups, with higher scores in the control group at both baseline (*p* < 0.001) and at 6-months (*p* < 0.001) (Table 5). At the 6-month examination about 34% of the patients in the test group and 27% in the control group showed a PI score of ≤ 10%, while corresponding proportion with a proximal PI score of ≥ 50% was 9% in the test and 19% in the control group.

### Multiple regression models

Two logistic regression models were formulated to predict subjective treatment outcome: Model I (Table 6) identified that ‘patients’ experiences of being involved in treatment’ was the only explanatory factor with a statistically significant impact on outcome in terms of overall satisfaction with the care provided, i.e. that treatment definitely was worth the cost, in terms of time, money and efforts (OR = 4.8; *p* < 0.001). Model II (Table 6) identified that subjective oral health outcome as very much/much better compared to before treatment, was significantly predicted by the ‘patient’s experiences of being involved in treatment’ (OR = 4.9; *p* < 0.001) and not being a smoker (OR = 0.45; *p* < 0.001). The level of explained variance (*R*^*2*^) of the two models was 19% and 16%, respectively.

**Table 6:** Multiple logistic regression models^†^(Enter) to predict patients’ (model 1) overall satisfaction with treatment, i.e. that the periodontal treatment was worth the cost, in terms of time, money and efforts (Definitely) and (model 2) patient reported satisfaction with oral health outcome of therapy compared to the way it was before (Very much better/Much better)

Variables	OR	95% CI	*p *value
Model 1: ‘treatment was worth the cost and efforts’			
Test group (ref: control group)	1.01	0.65–1.55	0.975
Current smoker (ref: non-smoker)	0.90	0.54–1.50	0.672
I have definitely been as involved as I wish in treatment (ref: else)	4.80	3.10–7.43	< 0.001
GOHAI, mean score at baseline	1.02	0.97–1.07	0.514
VAS, pain/discomfort during treatment	1.00	0.99–1.01	0.600
Model 2: ‘satisfaction with oral health outcome of therapy’			
Test group (ref: control group)	1.09	0.67–1.78	0.772
Current smoker (ref: non-smoker)	0.45	0.26–0.78	0.004
I have definitely been as involved as I wish in treatment (ref: else)	4.93	2.95–8.24	< 0.001
GOHAI, mean score at baseline	0.98	0.93–1.03	0.620
VAS, pain/discomfort during treatment	1.00	0.99–1.01	0.389

Model 1: n = 427. Hosmer and Lemeshow goodness of fit *x*^2^ = 4.94, degrees of freedom (d.f.) = 8, *p* = 0.76

Model 2: n = 428. Hosmer and Lemeshow goodness of fit *x*^2^ = 13.74, degrees of freedom (d.f.) = 8, *p* = 0.089

^†^Adjusted for background variables regarding, age, gender and education. Significance level of the models =  < 0.05

Finally, a linear regression model was formulated to predict clinical treatment outcome:

Model III (Table 7) identified that clinical outcome in terms of patients’ adherence to periodontal infection control, which was judged by the proximal BI scores at 6-months, was predicted by ‘patients’ experiences of being involved in treatment’ (*p* < 0.001) and proximal BI scores at baseline (*p* < 0.001). The explained variance (*R*^*2*^) of this model was 16%.

**Table 7:** Multiple linear regression model^†^ to predict treatment outcome in terms of patients’ adherence to self-performed infection control i.e. proximal bleeding score at 6-months examination

Variables	B	95% CI	*p *value
Constant	8.62	− 5.96–23.20	0.246
Test group (ref: control group)	− 0.23	− 3.47–3.02	0.891
Current smoker (ref: non-smoker)	1.76	− 2.12–5.63	0.373
I have definitely been as involved as I wish in treatment (ref: else)	− 6.62	− 10.01 to − 3.22	< 0.001
GOHAI, mean score at baseline	0.10	− 0.09–0.06	0.591
VAS, pain/discomfort during treatment	− 0.01	− 0.26–0.45	0.727
Proximal bleeding score at baseline	0.22	0.16–0.28	< 0.001

^†^Adjusted for background variables regarding, age, gender and education. n = 390. R^2^ = 0.16

ANOVA gives the global p-value of the model and the significances of the individual covariates are presented in the table

## Discussion

No substantial differences were found between the two treatment approaches, neither with regard to patient-reported experiences and outcomes, nor with regard to clinical outcome in terms of adherence to self-performed periodontal infection control at 6-months. Multiple regression models identified that the patient’s experiences of being involved in therapy decisions was a significant predictor for a desirable subjective and clinical treatment outcome. In addition, to be a current smoker counteracted the patient’s satisfaction with oral health outcome, while proximal BI scores at baseline predicted outcome in terms of proximal BI scores at 6-months.

To our knowledge, this is the first study focusing on patient-reported experiences and outcome measures following the GPIC approach, compared to CNST, performed by DHs in general dental praxis. Our hypothesis was that the GPIC approach, with its pronounced initial focus on patient education, should have benefits in terms of patient’s adherence to treatment regiments by means of self-performed periodontal infection control. Moreover, this pronounced initial focus on patient education might also be assumed to affect how patients experience the communication with the DH and their feelings of being involved in treatment and therapy decisions. The results did not confirm our assumptions and there were no significant differences between the two study groups, neither with regard to communicative aspects of therapy nor in any aspect related to outcomes in terms of oral hygiene behaviors, oral hygiene related self-efficacy or adherence to self-performed infection control at the 6-month evaluation.

The results of the current randomized field study are in line with previous RCT-studies and suggest that both non-surgical treatment approaches are equally effective to achieve periodontal infection control [9]. It should be noted, however, that the control group in the current study showed significantly higher PI scores at both baseline and at 6-month follow up after the CNST, than the test group. It could be speculated if subjects enrolled to the control group differed in some aspects compared to subjects enrolled to the test intervention. It could also be speculated if the DHs harbored some skepticism towards treatment in accord with the full-mouth approach, compared to how they usually perform the non-surgical periodontal therapy, that might have influenced which patient that were recruited to the respective treatment approaches. Since this was a field study, with randomization to treatment protocol based on DH-level, such bias in patient recruitment could not be ruled out. However, the study groups did not differ at baseline regarding individual characteristics, such as age, gender, education and smoking habits. In addition, there were no statistically significant differences between the two study groups at baseline regarding BI, i.e. primary clinical outcome, or with regard to self-reported oral health related problems as assessed with the GOHAI. Neither were there any statistically significant differences between the groups at baseline regarding the global question on self-perceived oral health, even though a tendency for a somewhat higher proportion among the controls that was dissatisfied with their oral health.

Treatment related pain or discomfort was rated on a low level on the VAS-scales, without any statistically significant differences between the two study groups. Even so, there was a tendency for a somewhat higher discomfort experienced in conjunction with the full-mouth instrumentation. A possible explanation for the results might be that the DHs offered less pain relief (anesthesia) in relation to the full-mouth therapy that might be something to keep in mind for the clinician. Still, the results are in line with previous RCT-studies indicating that the patients experience both treatment approaches as causing a low degree of pain/discomfort, without any significant differences between treatment approaches [12, 28].

A majority of the patients in both groups expressed that the communication with the DH during therapy had worked very well and most patients also considered that they have had the opportunity to be as involved as they wanted in therapy decisions. In addition, about 77% in both treatment groups judged their oral health as very much /much better after therapy and 63% of the patients in both groups considered that the treatment definitely had been worth the costs, in terms of time, money and efforts. Yet, a higher proportion of patients in the CNST group (10%, versus 5% in the GPIC group; *p* < 0.05) expressed that they were doubtful about if the treatment had been worth the cost. A possible explanation might be that more time was used for the CNST compared to the GPIC approach in the initial non-surgical treatment phase. However, time used for treatment was not analyzed in the current study. It has been argued that the GPIC approach offers tangible benefits for the patient compared to CNST since the guided approach is less time-consuming, i.e. fewer appointments and less chair time for treatment yet equally effective [12]. From the patients’ point of view the current results revealed an overall high satisfaction with both treatment approaches, but the results also indicate that the patients favored the GPIC approach regarding overall treatment costs.

Assessment with the GOHAI revealed a statistically significant change in mean scores from baseline to 6-months in both groups and with no significant difference between the groups, indicating less oral problems after non-surgical therapy, i.e. improved OHRQoL. The results are in line with previous studies showing that non-surgical periodontal therapy has a positive impact on oral problems and OHRQoL [13, 14, 29]. However, it should be noted that the GOHAI scores indicated that the current study sample had relatively minor/moderate oral problems already at baseline. In comparison with the study by Jönsson & Öhrn [14], involving a Swedish patient sample referred to a specialist clinic for treatment of periodontitis, the current study sample rated their oral problems at a somewhat lower level and this was specifically evident in relation to the GOHAI-items ‘worried or concerned’ and ‘nervous or self-conscious’. In both the current study and in the study by Jönsson & Öhrn [14] the GOHAI-items indicating least problems were ‘limit contacts with others’ and ‘uncomfortable eating with other people’.

In all three multiple regression models, patients’ experiences of ‘being involved in treatment’ was identified as a significant factor for a desirable treatment outcome. Hence, to feel as being part of treatment and therapy decisions predicted a high satisfaction with oral health after therapy compared to how it was before treatment, and also that treatment definitively was worth the cost and efforts. Moreover, to be involved was a significant predictor for patients’ adherence to self-performed periodontal infection control. In most of therapies, the patient’s adherence to treatment and treatment regiments is a key factor in order to reach desirable results. The basis for adherence is a good therapeutic alliance, which is built on a person-centered approach in therapy where the care-provider attaches importance of partnership and where the individual patient’s needs, situation and whishes are taken into account [30, 31]. Moreover, a good therapeutic alliance positively influences patient’s satisfaction with care [31]. The current results are thus in line with what previously reported in relation to other health care domains and also what has been emphasized in previous qualitative interview studies in dentistry, suggesting that the establishment of a positive therapeutic alliance are utmost important to reach desirable outcomes in the non-surgical periodontal therapy [20, 21, 32]. Still, adherence to health advice and treatment regiments is complex and there are of course several individual, disease and treatment factors that might influence outcome of therapy [33]. It is well known that smoking has a negative impact on periodontal health and outcome of periodontal therapy [34, 35] and in the current study smoking was also shown to counteract a desirable subjective oral health outcome. In addition, BI scores at baseline were a significant predictor of BI scores at 6-months. These results may partly be explained by disease severity and needs to be further analyzed in forthcoming studies. Nevertheless, the results of the linear multiple model elucidated the importance of a person-centered approach in interventions aiming to increase the patient’s motivation for beneficial oral-health behavioral efforts.

### Strengths and limitations

A strength of the current study is that it was performed in general dental practice including a large variety of professionals, clinics and patients, that is unique. Hence, even though robust evidence exists for the efficacy of the two non-surgical treatment approaches, this evidence is based on the results of RCT-studies performed under optimal controlled conditions with selected patient samples and often at specialist clinics. Since most of non-surgical treatments are performed in general practice it is utmost important to evaluate methods also in this context, i.e. *effectiveness* [8, 36, 37] and the current field study contribute with such important knowledge. Moreover, previous research had paid very little attention to how patients view the GPIC-approach in comparison with CNST [9] and thus, the current study contribute with valuable knowledge on such patient-reported experiences and outcomes of therapy. Strategies for quality control, including repeated site-visits by a project coordinator/monitor, were implemented and the findings are based on both patient-reported and clinical measures. The questionnaires/questions used were carefully chosen and tested for readability and understanding prior to the study. In addition, the internal drop-out rate in the questionnaires was low indicating that the patient’s considered the questions as understandable and relevant/worth to answer. Eighty-five percent in the test and 80% in the control group followed treatment per protocol to 6-months. Drop-outs were somewhat younger than those who followed treatment per protocol to 6-months (mean age 49 and 54 year; *p* < 0.001) but did not differ with regard to other individual baseline characteristics (number of teeth, gender, education and smoking habits). Additionally, there were no pertinent differences between the two study groups with regard to baseline characteristics and the reason behind the somewhat higher drop-out rate in the control group can only be speculated on. One possible explanation might be related to the results discussed earlier indicating a favor for the GPIC-approach regarding patients’ views on treatment costs and, as shown in Fig. 1, 13% in the control group failed to complete the initial, baseline phase of therapy compared to 8% in the test group. Still, considering the design of the study, the number of patients and therapists involved and with a long-term follow-up of 6-months, the participation rate must be considered as acceptable and there were no indication for that the small but differential drop-out rate between the study groups should constitute any potential bias for the current results [38, 39].

There are limitations of the current field study which need special mention. As discussed earlier some bias in patient recruitment to the respective study group could not be completely ruled out since randomization, for study practical reasons, was based on DH-level and it was the DH who decided which patient that was invited to the respective study protocol. In addition, for study practical reasons the same therapist who treated the patient also made all clinical measurements that is a weakness, but since the circumstances was the same for both study protocols this should not change the interpretation of the current results. Furthermore, even though all DHs were educated and trained for the study and repeatedly monitored during the study period, the study protocols were not always followed as intended. Hence, proper registrations of BI and PI scores were sometimes missed and the most common reason given for this was stress/lack of time. This was specifically evident regarding PI scores and sometimes the DH simply made a note in the chart about how she/he judged the patients oral hygiene. For that reason, PI scores was not included as an explanatory variable in the multiple linear regression model. Within the limitations of the current field study, however, the external validity of the results presented is high. Hence, the results of this large field study performed in general-every-day practice brings knowledge of value outside the research-setting that can be generalized to similar populations and clinical settings.

## Conclusion

In conclusion, the results suggest that no significant differences occur with regard to how patients experience therapy and outcome of the GPIC approach versus CNST. Regardless of treatment approach, the patient’s experiences of being involved in therapy decisions seem to be of high importance for satisfaction with care and for adherence to self-performed periodontal infection control.

## Data Availability

The datasets used and/or analyzed during the current study are available from the corresponding author on reasonable request.

## Abbreviations

GPIC: Guided periodontal infection control
CNST: Conventional non-surgical treatment
DH: Dental hygienist
RCT: Randomized controlled trail
OHRQoL: Oral health related quality of life
PRO: Patient-reported outcome
VG: The region of Västra Götaland
VAS: Visual analogue scale
GOHAI: The general oral health assessment index
BI: Bleeding score
PI: Plaque score
CI: Confidence intervals
PRE: Patient-reported experiences
OR: Odds ratio
